# Characterization of *Tamyb10* allelic variants and development of STS marker for pre-harvest sprouting resistance in Chinese bread wheat

**DOI:** 10.1007/s11032-016-0573-9

**Published:** 2016-11-05

**Authors:** Y. Wang, X. L. Wang, J. Y. Meng, Y. J. Zhang, Z. H. He, Y. Yang

**Affiliations:** 1 College of Life Sciences, Inner Mongolia Agricultural University, 306 Zhaowuda Road, Hohhot, Inner Mongolia 010018 China; 2 Institute of Crop Science, National Wheat Improvement Center, Chinese Academy of Agricultural Sciences (CAAS), 12 Zhongguancun South Street, Beijing, 100081 China

**Keywords:** Allelic variation, Germination index (GI), *Tamyb10-D1*, *Triticum aestivum*

## Abstract

Wheat grain color does not only affect the brightness of flour but also seed dormancy and pre-harvest sprouting (PHS) tolerance. The transcription factor *Tamyb10* is an important candidate for *R-1* gene, and the expression of its homologs determines wheat seed coat color. In the present study, the allelic variations of *Tamyb10* were explored in a set of Chinese bread wheat varieties and advanced lines with different PHS tolerance, and a sequenced-tagged site (STS) marker for *Tamyb10-D1* gene was developed, designated as *Tamyb10D*, which could be used as an efficient and reliable marker to evaluate the depth dormancy of wheat seeds. Using the marker *Tamyb10D*, 1629- and 1178-bp PCR fragments were amplified from the tolerant varieties, whereas a 1178-bp fragment was from the susceptible ones. Of the Chinese bread wheat varieties and advanced lines, 103 were used to validate the relationship between the polymorphic fragments of *Tamyb10D* and PHS tolerance. Statistical analysis indicated that *Tamyb10D* was significantly (*P* < 0.001) associated with depth of seed dormancy in these germplasms. To further confirm the association between allelic variants of *Tamyb10-D1* and PHS tolerance, 200 recombinant inbred lines (RILs) from the cross between Zhongyou 9507 (1178-bp fragment) and Yangxiaomai (1178- and 1629-bp fragments) were genotyped using the marker *Tamyb10D*. General linear model analysis indicated that variation in *Tamyb10-D1* had a significant (*P* < 0.001) association with the germination index (GI) values, explaining 13.7, 4.7, and 9.8 % of the phenotypic variation in GI in Shijiazhuang, Beijing, and the averaged data from those environments, respectively. In addition, among the 103 wheat varieties, 8 *Tamyb10* genotypes (*Tamybl0-A1*, *Tamybl0-B1*, and *Tamyb10-D1* loci) were detected, namely, aaa, aab, aba, abb, baa, bab, bba, and bbb, and these were significantly associated with GI value.

## Introduction

Pre-harvest sprouting (PHS) results in loss of grain weight and reduction in the end-use quality of kernels in cereals, especially in wheat. It is believed that PHS tolerance of wheat is predominantly attributed to seed dormancy (Bailey et al. 1999; Flintham 2000; Li et al. 2004; Gubler et al. 2005; Tan et al. 2006; Yang et al. 2007; Sun et al. 2012; Yang et al. 2014). Improvement of tolerance to PHS in wheat is a major breeding objective in China and many other countries such as Japan, Australia, Canada, and USA. Therefore, an understanding of genetic control of seed dormancy or PHS tolerance and development of functional markers are very important for marker-assisted breeding targeting for improvement of PHS tolerance in wheat.

Seed dormancy can be divided into embryo-imposed and coat-imposed dormancy. Many genes involved in seed dormancy are known to be involved in abscisic acid (ABA) synthesis and ABA signal transduction (Gubler et al. 2005). The viviparous (*Vp-1*) gene is an important regulator of late embryogenesis in maize and a regulator of late embryo development in bread wheat (McCarty et al. 1991). *AtABI3* in *Arabidopsis* is an orthologous gene of *ZmVp-1* in maize and *OsVp-1* in rice (Koornneef et al. 1989; McCarty et al. 1991; Hattori et al. 1994). Furthermore, the *AtLEC2*, *AtDOG1*, *AtHUB1*, and *KYP/SUVH4* genes are also important for controlling seed dormancy (Stone et al. 2001; Bentsink et al. 2006; Liu et al. 2007; Zheng et al. 2012). In wheat, *TaSdr* genes were cloned and proved to be associated with tolerance to PHS in bread wheat (Zhang et al. 2014). Moreover, the *TaVp*-*1* genes involved in ABA signal transduction are also important seed dormancy-related transcription factors, and a positive correlation was present between seed dormancy and embryo sensitivity to ABA (Nakamura and Toyama 2001; McKibbin et al. 2002). Six alleles of *TaVp*-*1B* were identified, designated as *TaVp*-*1Ba*, *TaVp*-*1Bb*, *TaVp*-*1Bc*, *TaVp*-*1Bd*, *TaVp*-*1Be*, and *TaVp*-*1Bf*, respectively (Yang et al. 2007; Xia et al. 2008; Yang et al. 2009; Chang et al. 2010). Based on the allelic variation, a STS marker Vp1B3 associated with seed dormancy was developed; variations with *TaVp*-*1Bb* and *TaVp*-*1Bc* were associated with higher PHS tolerance (Yang et al. 2007). For *TaVp*-*1A*, rich allelic variations were detected (Chang et al. 2011; Sun et al. 2012; Yang et al. 2014) and another STS marker Vp1A3 for PHS tolerance was developed; the variations with the allele combinations *TaVp-1Agm*/*TaVp-1Bb*, *TaVp-1Agm*/*TaVp-1Ba*, *TaVp-1Aim*/*TaVp-1Bb*, and *TaVp-1Aam*/*TaVp-1Bb* showed higher PHS resistance (Yang et al. 2014).

The dormancy of lighter colored seed was weaker than that of darker colored seed in *Arabidopsis* (Debeaujon et al. 2000), indicating the association of grain dormancy with grain color. Wheat grain color is controlled by *R-1* genes located in the distal region of the long arms of wheat chromosomes 3A, 3B, and 3D (*R-A1*, *R-B1*, and *R-D1*, respectively). The recessive alleles for white grains have been denoted *R-A1a*, *R-B1a*, and *R-D1a*, while the dominant alleles for red grains, *R-A1b*, *R-B1b*, and *R-D1b*, respectively. For dominant alleles *R-A1b*, *R-B1b*, and *R-D1b*, any one is sufficient to result in red grains, and grain’s redness increases in a gene dosage-dependent manner (McIntosh et al. 2010). The *R-1* genes affect the sensitivity of embryos to ABA and the development of grain dormancy. It was proposed that one of the Myb-type genes of *Arabidopsis*, AtMYB2, might be involved in ABA signal transduction (Abe et al. 2003).

The pigment of red grain color is composed of anthocyan in anidin and catechin, which are synthesized by the enzymes chalcone synthase (CHS), chalcone flavanone isomerase (CHI), flavanone3-hydroxylase (F3H), and dihydroflavonol-4-reductase (DFR) in the flavonoid synthesis pathway (Holton and Cornish 1995; Chopra et al. 1996; Mol et al. 1998). Anthocyanins, phlobaphenes, flavonols, and proanthocyanidins (PAs) are all synthesized through the same early flavonoid biosynthetic pathway branched out into the individual pathway. CHS, CHI, F3H, and DFR are expressed mainly in the immature red grains and are almost totally suppressed in white grains (Himi and Noda 2005). In addition, several regulatory proteins involved in flavonoid biosynthesis have been reported in various species, such as maize, petunia, snapdragon, and *Arabidopsis* (Winkel-Shirley 2001; Mol et al. 1998). Two types of transcription factors grouped as the R/B family (basic helix-loop-helix (bHLH)-type) and the *C1*/*Pl* family (Myb-type) were able to upregulate all the structural genes required for the production of anthocyanin. *Myb/c1* are transcriptional activators of flavonoid synthesis genes (Himi and Noda 2005), approximately 30 cM proximal to the *Vp1* locus, consistent with observed linkage between grain dormancy and red grain (Groos et al. 2002; Himi and Noda 2004). *Vp-1* is a key element that plays an important role in the seed maturation processes, such as seed dormancy and seed desiccation (McCarty et al. 1991; Giraudat et al. 1992). It was reported that *Vp1*/ABA/GA coordinated the control of grain color and PHS via Myb-dependent and Myb-independent pathways (Xia et al. 2009). Mutations in *Vp-1* inhibit anthocyanin in synthesis (Robertson 1955; McCarty et al. 1989). *Vp-1* interacts with the Sph cis-element in the promoter region of the Myb/c1 gene and regulates its expression (Hattori et al. 1992; Carson et al. 1997).

*Hvmyb10* was also a key factor of grain dormancy in barley (Himi et al. 2012). *Tamyb10- A1*, *Tamyb10-B1*, and *Tamyb10-D1* genes, located on chromosomes 3A, 3B, and 3D in wheat, respectively, encode R2R3-type MYB domain proteins, similar to TT2 of *Arabidopsis* that controls PA synthesis and induces the expression of flavonoid biosynthetic genes such as *CHS*, *CHI*, *F3H*, and *DFR*, which are also essential for the synthesis of both anthocyanins and proanthocyanidins (Himi et al. 2011). In addition, *Tamyb10* was demonstrated to activate anthocyanin biosynthesis genes with synergy of the bHLH-type protein through a transient assay, and it is likely to be a strong candidate for the *R-1* gene of wheat, which regulates wheat grain color (Himi et al. 2011); the red-grained wheat varieties are usually more tolerant to PHS than white-grained wheat varieties (Flintham 2000; Warner et al. 2000; Himi et al. 2002). However, not all the red-grained wheat varieties are higher PHS resistant than white grained, and *Tamyb10-A1* also expressed in white-grained EMS-AUS in immature grain (DPA 5) (Himi et al. 2011), indicating that expression of *Tamyb10* may play an important role in interacting with *Vp-1* in PHS tolerance mechanism in white-grained wheat. In our previous study, some landraces, such as Suiningtuotuomai (average GI = 0.10) and Waitoubai (average GI = 0.07), had the strong PHS resistance, but they did not carry any of the PHS-resistant allele combinations of *TaVp-1Agm*/*TaVp-1Bb*, *TaVp-1Agm*/*TaVp-1Ba*, *TaVp-1Aim*/*TaVp-1Bb*, and *TaVp-1Aam*/*TaVp-1Bb* (Yang et al. 2014), which might lay in the fact that there were still other factors affecting PHS except for *Vp-1* gene.

The objectives of the present study were to identify the allelic variations at *Tamyb10* locus among Chinese wheat varieties and advanced lines with different level of PHS tolerance and develop efficient markers for marker-assisted breeding. Furthermore, the identification of these new *Tamyb10* resources could also contribute to our understanding of the mechanisms underlying seed dormancy or PHS tolerance in bread wheat.

## Materials and methods

### Plant materials

Ten bread wheat varieties were used for PCR amplification of *Tamyb10-A1*, *Tamyb10-B1*, and *Tamyb10-D1*. There were five PHS-resistant varieties, Xiaobaiyuhua, Yumai 18, Yangxiaomai, Xiaoyuhua, and Xiaoye 6, with the germination index (GI) values of 0.04, 0.07, 0.08, 0.10, and 0.14, respectively, and five PHS-susceptible varieties, Zhou 8425B, Jimai 19, Jing 411, Hengshui 7228, and Zhongyou 9507, with the GI values of 0.56, 0.58, 0.64, 0.68, and 0.71, respectively.

In total, 103 Chinese varieties and advanced lines, with different PHS resistance from the China Autumn-sown Wheat Region (CAWR), representing more than 85 % of wheat production areas in China, were used for association study as presented in Table 1. Among these, 18 varieties had a GI less than 0.15 and 85 between 0.15 and 0.71. The GI was determined based on the average data across two cropping seasons at two locations, Anyang in Hennan Province and Beijing (Table 1) (Yang et al. 2014). Moreover, 200 recombinant inbred lines (RILs), derived from the Yangxiaomai/Zhongyou 9507 cross, were used to confirm relationship between the allelic variations in *Tamyb10-D1* and PHS tolerance; Yangxiaomai is a Chinese landrace and had a low GI value (0.08), whereas Zhongyou 9507 had a high GI value (0.71).

**Table 1:** GI values and *Tamyb10* alleles in 103 Chinese bread wheats

Number	Cultivar	GI (%)	*Tamyb10-A1*	*Tamyb10-B1*	*Tamyb10-D1*	Genotype
1	Zhongyou 9507	0.71	a	b	a	aba
2	CA0459	0.71	a	b	a	aba
3	CA0474	0.70	b	b	a	bba
4	Hengshui 7228	0.69	a	a	a	aaa
5	Han 6172	0.67	a	b	a	aba
6	CA0349	0.67	b	b	a	bba
7	Zhenmai 98	0.66	a	b	a	aba
8	Baiyingdong 2	0.65	a	b	a	aba
9	Jing 411	0.64	a	a	a	aaa
10	CA0475	0.63	b	b	a	bba
11	Jinai 16	0.62	b	b	a	bba
12	CA0306	0.62	b	b	b	bbb
13	Hongliang 4	0.60	b	b	a	bba
14	Xiaoyan 22	0.59	a	b	a	aba
15	Jimai 19	0.58	b	b	a	bba
16	Taishan 008	0.56	a	b	a	aba
17	Zhou 8425B	0.56	a	a	b	aab
18	Shijiazhuang 8	0.56	a	b	a	aba
19	Yannong 19	0.54	b	b	a	bba
20	CA0465	0.54	b	b	a	bba
21	Jishi 02–1	0.52	a	b	a	aba
22	Hongsuibai	0.52	b	b	b	bbb
23	Zhenmai 004	0.51	a	b	a	aba
24	Hengguan 35	0.51	b	b	a	bba
25	Yumai 2	0.51	a	b	a	aba
26	Lumai 14	0.50	a	b	a	aba
27	Zhoumai 16	0.49	a	b	a	aba
28	CA0420	0.49	b	b	a	bba
29	Shan 213	0.49	a	b	a	aba
30	Han 5316	0.48	b	b	a	bba
31	Jinmai 5	0.47	a	b	a	aba
32	Xinmai 9	0.47	a	b	a	aba
33	Taishan 9818	0.47	b	b	a	bba
34	CA0471	0.46	b	a	a	baa
35	CA0175	0.45	a	b	a	aba
36	Yumai 34	0.45	b	b	a	bba
37	Tutoumai(jia)	0.44	a	b	a	aba
38	Zhongyu 6	0.43	b	b	a	bba
39	Shi 4185	0.43	a	b	a	aba
40	Huaimai 20	0.43	b	b	a	bba
41	Xinong 88	0.42	b	b	a	bba
42	Gaocheng 8901	0.42	a	a	b	aab
43	Yanfu 188	0.42	a	b	a	aba
44	Xumai 856	0.42	a	b	a	aba
45	Yanshi 4110	0.41	a	b	b	abb
46	Zimai 12	0.40	a	a	a	aaa
47	Jinan17	0.40	b	b	a	bba
48	Baiyuhua	0.39	b	b	a	bba
49	Xinmai 18	0.38	a	b	b	abb
50	Weimai 8	0.38	a	b	a	aba
51	Yannong 15	0.37	b	a	a	baa
52	Zheng 9023	0.37	b	a	a	baa
53	Shan 160	0.37	a	a	a	aaa
54	CA0481	0.37	b	a	a	baa
55	Yumai 47	0.37	a	a	a	aaa
56	Heng 95Gian 26	0.37	a	a	a	aaa
57	Han 3475	0.37	a	b	a	aba
58	Xinmai 11	0.36	a	a	a	aaa
59	Zhongyu 5	0.36	a	a	a	aaa
60	Yanzhan 1	0.33	a	a	a	aaa
61	Xinong 979	0.33	a	a	a	aaa
62	Linmai 2	0.33	a	a	b	aab
63	Liangxin 99	0.33	b	a	b	bab
64	Lumai 21	0.32	a	a	a	aaa
65	Shixin 733	0.31	b	a	a	baa
66	Kenong 9204	0.30	a	a	a	aaa
67	Huixianhong	0.29	b	a	b	bab
68	PH82–2	0.29	a	a	a	aaa
69	Baisuibai	0.25	b	a	a	baa
70	Shanyou 225	0.25	b	a	a	baa
71	CA0178	0.24	b	a	a	baa
72	Yinbinbaimaizi	0.24	a	a	b	aab
73	Zheng 366	0.24	a	a	a	aaa
74	Zhoumai 19	0.24	b	a	a	baa
75	Neixiang 19	0.24	b	a	a	baa
76	Yongchuanbaimaizi	0.23	a	a	b	aab
77	Bainong 64	0.23	a	b	a	aba
78	Shan 253	0.23	a	a	a	aaa
79	Pumai 9	0.20	b	a	a	baa
80	Xiaoyan 54	0.19	a	a	a	aaa
81	Jimai 21	0.18	a	a	a	aaa
82	Shannong 757	0.17	a	a	a	aaa
83	Nvermai	0.16	a	a	b	aab
84	Aikang 58	0.15	a	a	a	aaa
85	Lankao 906	0.15	a	a	a	aaa
86	Xiaoyan 6	0.14	a	a	a	aaa
87	Rongchangbaimaizi	0.10	b	b	b	bbb
88	Suiningtuotuomai	0.10	b	a	b	bab
89	Xiaoyuhua	0.10	a	a	a	aaa
90	Tuhulutou	0.09	a	a	b	aab
91	Langzhongbaimai	0.08	a	a	b	aab
92	Neixiang 173	0.08	b	a	a	baa
93	Wanxianbaimai	0.08	a	a	b	aab
94	Yangxiaomai	0.08	b	a	b	bab
95	Waitoubai	0.07	a	a	b	aab
96	Yumai 18	0.07	a	a	a	aaa
97	Xinmai13	0.07	a	a	a	aaa
98	Peilingxuxubaimai	0.07	a	a	b	aab
99	Xuyong	0.07	b	a	b	bab
100	Chuan 362	0.05	a	a	b	aab
101	Xiaobaiyuhua	0.04	a	b	b	abb
102	Fengchan 3	0.04	a	a	a	aaa
103	Xumai 954	0.04	a	a	a	aaa

### Primer design

Nine gene-specific primers, Tamyb10-AF_1_/R_1_, Tamyb10-AF_2_/R_2_, Tamyb10-AF_3_/R_3_, Tamyb10-BF_1_/R_1_, Tamyb10-BF_2_/R_2_, Tamyb10-BF_3_/R_3_, Tamyb10-DF_1_/R_1_, Tamyb10-DF_1_/R_2_, and Tamyb10-DF_3_/R_3_, were used to amplify the *Tamyb10-A1*, *Tamyb10-B1*, and *Tamyb10-D1* genes, respectively (Table 2). The other primer sets Myb10-A1, Myb10-A1, Myb10-A1, and Myb10-B were *Tamyb10*-specific markers used for determining the allelic variants of *Tamyb10* (Himi et al. 2011).

**Table 2:** Primer sets for cloning *Tamyb10* genes and characterization of *Tamyb10* allelic variants in this study

Primer set	Primer sequence	Anneal temperature (°C)	Fragment size (bp)
Tamyb10-A F1	ATGGCTGCTCCCAAAGCTCTCA	63.6	1948 bp
Tamyb10-A R1	CGATGAGCTCCTCTTCGTCGTT	61.7	1948 bp
Tamyb10-A F2	AATCGCTGCGGTAAGAGCTG	59.9	1082 bp
Tamyb10-A R2	GCAGCATCCTCTTGCTCAGG	60.8	1082 bp
Tamyb10-A F3	TCAAGAACTACTGGAACACC	54.1	540 bp
Tamyb10-A R3	CGTATTTTACTGCACGTAAC	52.3	540 bp
Tamyb10-B F1	ATGGGGAGGAAACCATGCTG	59.2	447 bp
Tamyb10-B R1	CCGGCAGCTCTTTCCGCAC	63.6	447 bp
Tamyb10-B F2	AATCGGTGCGGAAAGAGCTG	60.1	1198 bp
Tamyb10-B R2	CCGTATCGGGCTGCTGCTC	62.2	1198 bp
Tamyb10-B F3	TGCCGGGGCGAACAGACAAT	63.9	515 bp
Tamyb10-B R3	TGTCACCCGGGCCATCAAAG	62.2	515 bp
Tamyb10-D F1	ATGGGGAGGAAGCCATGCTG	61.4	1419 bp
Tamyb10-D R1	CGGTCACTGTTATCTGACGCTGGAT	64.4	1419 bp
Tamyb10-D F1	ATGGGGAGGAAGCCATGCTG	61.4	1629 bp
Tamyb10-D R2	ACTGCTGCTCGTGCCCTCC	63.6	1629 bp
Tamyb10-D F3	GGGCGAACAGACAATGAGAT	57.3	630 bp
Tamyb10-D R3	CTTTGTTTACAGCACCAC	51.0	630 bp
Myb10-A1 F	CTATGTGGATGGCCTTGCAT	57.2	665 bp
Myb10-A1 R	CTACCAGCTCGTTTGGGAAG	57.7	665 bp
Myb10-A2 F	TTTCAATCGAGTGGGCATAA	54.3	536 bp
Myb10-A2 R	CCTGACGATGAGCTCCTCTT	58.0	536 bp
Myb10-A3 F	TCCCTACATGGGAGACAGAGA	58.4	565 or 2750 bp
Myb10-A3 R	TGTTATCACATGCTGATCCTGA	56.8	565 or 2750 bp
Myb10-B F	AGCAAGAGGAACCTGCAGTC	59.3	262 or 282 bp
Myb10-B R	GATGCCCTCCAGATCAAGGT	58.5	262 or 282 bp

### DNA extraction and PCR amplification

Genomic DNA was extracted from seedlings using the method described by Gale et al. (2001). PCR reactions were performed in an Applied Biosystems 2720 thermal cycler in a total volume of 25 μl, including 2.5 μl 10× PCR buffer, 125 μM of each dNTP, 4 pmol of each primer, 1.0 U of TaKaRa La*Taq* polymerase, and 50 ng of template DNA. PCR amplification were 94 °C for 5 min, followed by 35 cycles of 94 °C for 1 min, 53 °C–68 °C for 45 s, and 72 °C for 1.5 min, with a final extension of 72 °C for 10 min. Amplified PCR fragments were separated on 1.5 % agarose gel.

### DNA sequencing

The PCR products were sequenced from both strands by Huada Gene Biological Technology Co. Ltd. (http://www.genomics.cn/index.php). Sequence analysis and characterization were performed using software DNAMAN (http://www.lynon.com).

### Statistical analysis

Analysis of variance was conducted by PROC MIXED in the Statistical Analysis System (SAS Institute, 8.0) with genotype clusters indicated by two types of fragments, which were amplified with STS marker *Tamyb10D*, as a categorical variable to derive mean GI value from each cluster and to test significant levels. The genotype clusters were treated as fixed effects, while genotypes nested in clusters and years were treated as random. Pearson’s linear correlation coefficients for GI between years were obtained by SAS PROC CORR. Significance of the association between *Tamyb10-D1* and PHS tolerance in the RIL population was evaluated using general linear model (GLM) analysis, based on the phenotypic variation in GI explained by *Tamyb10D* estimated.

## Results

### Amplification and sequence analysis of three *Tamyb10* homologs in varieties with different PHS tolerance

Full sequences of three *Tamyb10* homologs were isolated using genome-specific primers (Table 2). Firstly, *Tamyb10-A1* was amplified with the primer sets Tamyb10-AF_1_/R_1_, Tamyb10-AF_2_/R_2_, and Tamyb10-AF_3_/R_3_ in 10 bread wheats with different PHS tolerance. Sequence alignment showed no difference among the 10 bread wheat varieties, but the full sequence of these wheat varieties had 12 SNPs compared with the *Tamyb10-A1* (AB191458) (2 SNPs located in exons and 10 in introns); for two SNPs in exons, one G to T change at position 177 bp in the first exon did not induce any change of amino acids, whereas the other G to A at position 2957 bp in the third exon induced the change of amino acid. The other 10 SNPs were located in the first and second introns. In addition, two 1-bp insertions (T and A) were found in the position of 1528 and 2354 bp in the introns.

The *Tamyb10-B1* was amplified from the 10 bread wheats with the primer sets Tamyb10-BF_1_/R_1_, Tamyb10-BF_2_/R_2_, and Tamyb10-BF_3_/R_3_, respectively. For the PCR fragment amplified with primer sets Tamyb10-BF_1_/R_1_ and Tamyb10-BF_3_/R_3_, the sequence alignment showed that cultivar Jimai 19 had very similar sequence with Yangxiaomai except that Jimai 19 has two 6-bp insertions located in 368 and 399 bp, respectively. In addition, compared with the *Tamyb10-B1* (AB191459.1), the sequence stitching amplified with primer set Tamyb10-BF_1_/R_1_ and Tamyb10-BF_2_/R_2_, in 10 bread wheat varieties, had 5 SNPs and 2-bp (GT) insertion, of which 2 were located in the first exon, i.e., G to A at position 41 bp that did not induce any change of amino acid and A to T at position 179 bp, while other three located in the introns, i.e., A to T at position 179 bp, A to G at position 685 bp, and C to T at position 1147 bp. The 2-bp insertion (GT) at position 286 bp was also located in the first intron. Polymorphic fragment was detected in the 10 bread wheats with different PHS tolerance amplified with primer set Tamyb10-BF_3_/R_3_. A 534-bp fragment was amplified in varieties Xiaobaiyuhua, Jimai 19, and Zhongyou 9507, whereas a 515-bp fragment was detected in the other varieties. Sequence alignment showed a 19-bp deletion in exon 2 of *Tamyb10-Ba*, comprising five GCC repeats and four bases of GACG, and caused a frame shift in its open-reading frame. This result was described in Himi et al. (2011); compared with *Tamyb10-Ba*, *Tamyb10-Bb* had a 19-bp insertion. Therefore, varieties Xiaobaiyuhua, Jimai 19, and Zhongyou 9507 amplified with a 534-bp fragment had allele *Tamyb10-Bb*, while the others amplified with a 515-bp fragment were *Tamyb10-Ba*.

**Fig. 1:**
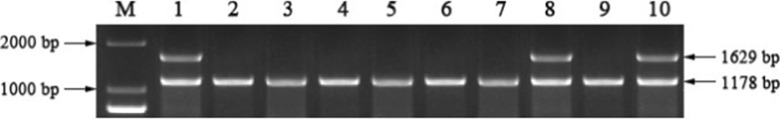
PCR fragments amplified with Tamyb10-DF_1_/R_2_ in 10 Chinese wheat varieties with different PHS tolerance. The full sequence of *Tamyb10-D* had been subcloned in varieties Zhou 8425B, Xiaobaiyuhua, and Yangxiaomai with the primer sets Tamyb10-DF_1_/R_2_. *M* Trans 2 k, *1* Zhou 8425B (average germination index 0.56), *2* Jimai 19 (0.58), *3* Jing 411(0.64), *4* Zhongyou 9507 (0.71), *5* Hengshui 7228 (0.68), *6* Yumai 18 (0.07), *7* Xiaoyuhua (0.10), *8* Xiaobaiyuhua (0.04), *9* Xiaoyan 6 (0.14), *10* Yangxiaomai (0.08). Note that the *number in the bracket* indicates the average GI values

The full sequence of *Tamyb10-D1* was amplified from the 10 bread wheats with the genome-specific primer sets Tamyb10-DF_1_/R_1_, Tamyb10-DF_1_/R_2_, and Tamyb10-DF_3_/R_3_. The polymorphism was detected from the PCR products of primer sets Tamyb10-DF_1_/R_2_ and Tamyb10-DF_3_/R_3_ in agarose gel but not from Tamyb10-DF_1_/R_1_. A fragment (1419 bp) of *Tamyb10-D1* was amplified in the 10 bread wheats by the primer set Tamyb10-DF_1_/R_1_. Compared with the *Tamyb10-Db* (AB191460, red-grained wheat Chinese spring), the part sequence of *Tamyb10-D1* had five SNPs; among them, G to A at position 2160 bp was located in the first exon that changed the triplet code AGA (polar uncharged amino acid glycine) into AAA (nonpolar alanine), while the other four, C to T, G to A, T to C, and T to A at positions 2637, 2698, 2823, and 3220 bp, respectively, were located in the second intron. Two kinds of fragments were detected with the primer set Tamyb10-DF_1_/R_2_. A 1629- and a 1178-bp fragment were amplified in Zhou8425B, Xiaobaiyuhua, and Yangxiaomai, whereas only a 1178-bp fragment was amplified in the other seven varieties; sequence alignment showed that the 1178-bp fragment was nonspecific amplification product, but the 1629-bp fragment was specific amplification fragment (Fig. 1). For the primer set Tamyb10-DF_3_/R_3_, the specific PCR products were obtained from varieties Zhou 8425B, Xiaobaiyuhua, and Yangxiaomai, but no PCR fragment product was detected from the other seven.

The full sequence of *Tamyb10-D1* was amplified in Zhou 8425B, Xiaobaiyuhua, and Yangxiaomai, which had 99.58 % similarity with that of *Tamyb10-Db*; therefore, varieties Zhou 8425B, Xiaobaiyuhua, and Yangxiaomai had the allele *Tamyb10-Db*. In the other seven varieties, no specific PCR fragments were detected with the primer sets Tamyb10-DF_1_/R_2_ and Tamyb10-DF_3_/R_3_; these varieties had the allele *Tamyb10-Da*, in which just a 1419-bp fragment was amplified with the primer set Tamyb10-DF_1_/R_1_.

### Development and validation of *Tamyb10D* STS marker for PHS resistance

**Fig. 2:**
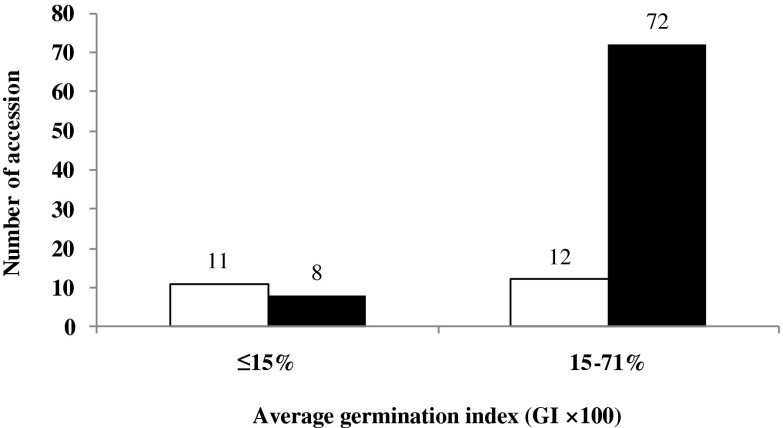
Association between PHS tolerance (germination rate) and the size of PCR fragments amplified with *Tamyb10D* in 103 white-grained bread wheat varieties. The *white columns* indicate the number of accessions with 1629 bp, and the *black columns* indicate the number of accessions with no specific amplified fragment

Based on the sequence analysis, a STS marker of *Tamyb10-D1*, designated as *Tamyb10D* (primer set Tamyb10-DF_1_/R_2_), was developed and used for association analysis with 103 Chinese varieties and advanced lines. The PCR amplification indicated two types of fragments (1629- and 1178-bp fragments and 1178-bp fragment) amplified with primer set Tamyb10-DF_1_/R_2_ (Table 2 and Fig. 1). Among the 103 varieties and lines tested, 23 were the genotype of *Tamyb10-D1b* amplified with 1629- and 1178-bp fragments, whereas 80 had *Tamyb10-D1a* with only one 1178-bp fragment (Table 1 and Fig. 2), which accounted for 22.3 and 77.7 %, and had an average GI values of 0.245 and 0.398, respectively. The GI values of the 103 varieties were consistent over the 2 years (*r* = 0.966, *P* < 0.0001), with mean value and standard deviation being 0.361 ± 0.2 in 2006 and 0.359 ± 0.19 in 2007, respectively. Analysis of variance in *Tamyb10-D1* indicated significant differences between two clusters for GI (*P* = 0.00012). The genotypes with 1178-bp fragment were more susceptible to PHS with an average GI value of 0.398, compared with that of 1178- and 1629-bp fragments, showing more tolerance to PHS with an average GI value of 0.245. This indicated that the genotypes with the 1178- and 1629-bp fragments were more resistant to PHS than those with the 1178-bp fragment.

**Fig. 3:**
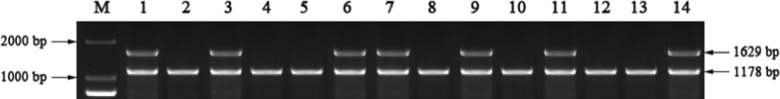
Detection of allelic variation of *Tamyb10-D1* in RIL alleles including 1629 bp (Yangxiaomai) and no specific PCR product (Zhongyou 9507) are marked with the *lanes 1–2*, respectively. *Lanes 3–14* are the RILs developed from cross between Yangxiaomai and Zhongyou 9507

To further confirm the association, a RIL population developed from the cross of Yangxiaomai/Zhongyou 9507 was genotyped using *Tamyb10D* (Fig. 3). Statistical analysis confirmed the significant association (*P* < 0.001) of allelic variations of *Tamyb10-Db* with GI value and PHS tolerance. In this population, *Tamyb10-D1* gene explained 13.7, 4.7, and 9.8 % of the phenotypic variations in Shijiazhuang, Beijing, and the averaged data from those environments, respectively, based on the test of STS marker *Tamyb10D* (Table 3).

**Table 3:** Association analysis between *Tamyb10D* and GI values in 200 RILs using GIM model

Trait	*Tamyb10D*	No. of alleles	Mean of phenotype (%)	*F* value	Explanation of phenotypic variation (*R* ^2^) (%)
Shijiazhuang GI values	a type of fragment	125	9.49	32.497*	13.7
	b type of fragment	75	22.35		
Beijing GI values	a type of fragment	125	28.99	10.804*	4.7
	b type of fragment	75	39.39		
Average GI values	a type of fragment	125	19.24	22.567*	9.8
	b type of fragment	75	30.87		

*Significant association between *Tamyb10D* and phenotypic variation at the 0.001 level

### Characterization of *Tamyb10* allelic variants

**Fig. 4:**
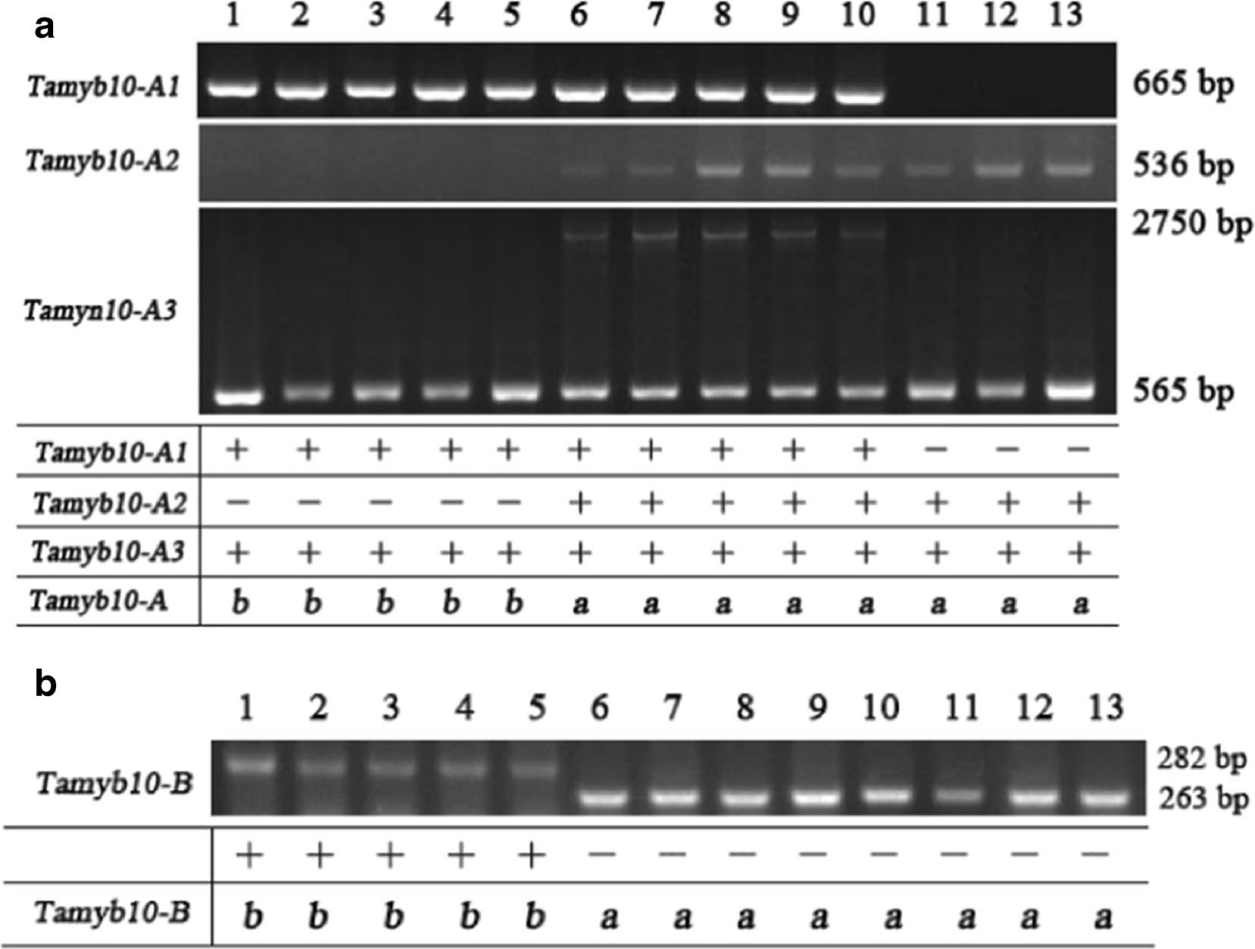
Identification of haplotypes of *Tamyb10* in part wheat varieties using four pairs of specific primers. *1* Hengguan 35, *2* Yannong 19, *3* CA0420, *4* Xinong 88, *5* Han 5316, *6* Lumai 21, *7* Nvermai, *8* Chuan 362, *9* Shan 253, *10* PH82–2, *11* Zheng 366, *12* Yumai 47, *13* Waitoubai

The allelic variants of *Tamyb10-A1* and *Tamyb10-B1* were identified with the primer sets Myb10-A1, Myb10-A2, Myb10-A3, and Myb10-B (Himi et al. 2011). The alleles of *Tamyb10-D1* were detected with the primer set Tamyb10-DF_1_/R_2_. Firstly, three primer sets Myb10-A1, Myb10-A2, and Myb10-A3 were used to identify the allelic variants of *Tamyb10-A1* in the 103 varieties with different dormancy level. Thirty-eight *Tamyb10-A1b* varieties showed a 665-bp fragment with primer set Myb10-A1 and a 565-bp fragment with primer set Myb10-A3 but not with primer set Myb10-A2 (Table 4 and Fig. 4a). Sixty-five *Tamyb10-A1a* varieties showed a 665-bp fragment with primer set Myb10-A1, 636-bp fragment with primer set Myb10-A2, and 565- or 565- and 2750-bp fragments with primer set Myb10-A3 (Table 4 and Fig. 4a). Among them, five varieties including Lumai 21, Nvermai, Chuan 362, Shan 253, and PH82–2 had the heterozygote fragments with 2750 and 565 bp using the primer set Myb10-A3. Other three *Tamyb10-A1a* varieties Zheng 366, Yumai 47, and Waitoubai showed amplified fragments with primer sets Myb10-A2 and Myb10-A3 but not with primer set Myb10-A1 (Fig. 4a). Thirty-eight *Tamyb10-A1b* varieties had mean GI value and standard deviation of 0.390 ± 0.028, and 65 *Tamyb10-A1a* varieties had mean GI value and standard deviation of 0.342 ± 0.024, and analysis of variance indicated that there were not significant differences between two clusters for GI.

**Table 4:** Distribution of different *Tamyb10* alleles in 103 white-grained wheat varieties

PHS resistance	No. of varieties	GI (%)	GI range (%)	*Tamyb10-A1*		*Tamyb10-B1*		*Tamyb10-D1*	
				b	a	b	a	b	a
Resistant	35	14.1	0–26	11	24	3	32	14	21
Moderately resistant	56	43.2	27–60	22	34	35	21	8	48
Susceptible	12	66.2	61–100	5	7	10	2	1	11
Total	103			38	65	48	55	23	80

Germination index (GI) is the mean of two locations over 2 years

The primer set Myb10-B was used to identify the allelic variants of *Tamyb10-B1* in these germplasm. Two types of fragments (282 and 263 bp) were detected in 103 varieties (Fig. 4b). Genotypes with a 282-bp fragment were the allele *Tamyb10-B1b*, whereas genotypes with 263 bp were *Tamyb10-B1a*. Forty-eight *Tamyb10-B1b* varieties had mean GI value and standard deviation of 0.491 ± 0.020 and 55 *Tamyb10-B1a* varieties had mean GI value and standard deviation of 0.245 ± 0.020 (Table 4), and analysis of variance indicated significant differences between two clusters for GI (*P* = 0.9 × 10^−13^). The data showed that the allelic variants of *Tamyb10-B1* were associated with PHS tolerance.

With the marker *Tamyb10D*, a 1629-bp fragment was amplified in genotypes of *Tamyb10-D1b*, while nonspecific amplification was found in the genotype *Tamyb10-Da*. Twenty-three *Tamyb10-D1b* varieties and 80 *Tamyb10-D1a* varieties were detected (Fig. 1 and Table 4).

**Fig. 5:**
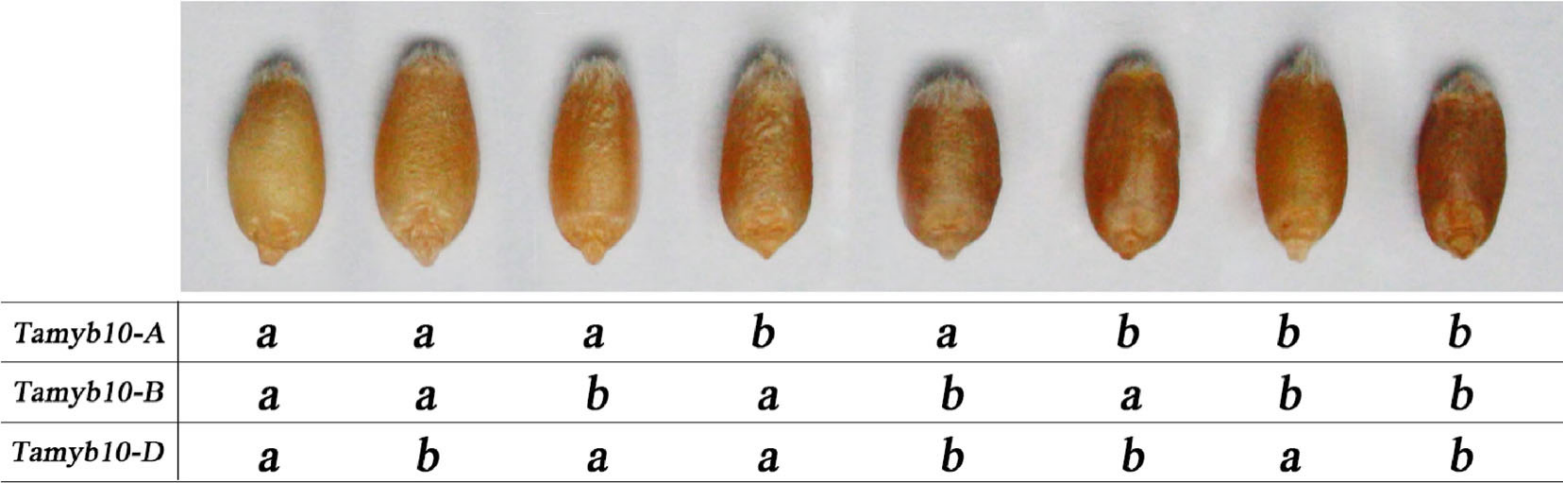
Grain color of genotypes with different *Tamyb10* allele combinations. From *left to right*, the names of varieties were Xiaoyan 6, Zhou 8425B, Zhongyou 9507, Xiaobaiyuhua, Zhoumai 19, Yangxiaomai, Jimai 19, and Hongsuibai, respectively

In the 103 Chinese bread wheats with different PHS tolerance, 8 *Tamyb10* genotypes (*Tamyb10-A1*, *Tamyb10-B1*, and *Tamyb10-D1* loci) were detected, namely, aaa, aab, aba, abb, baa, bab, bba, and bbb, which showed a continuous color change with increase of dominant allele “b” (Fig. 5). Multiple comparisons indicated that the average GI was significantly different among genotypes with different allele combinations such as aaa and aba, aaa and bba, aab and aba, aab and bba, aba and abb, aba and bab, abb and bba, baa and bba, and bab and bba (*P* < 0.01). As shown in Table 5, the genotypes with the allele combinations aaa, aab, abb, baa, and bab showed resistance to PHS with average GI values of 0.264, 0.198, 0.275, 0.283, and 0.172, respectively, whereas the genotypes with aba and bba showed susceptible to PHS with average GI values of 0.508 and 0.519, respectively (Table 5).

**Table 5:** Germination index (GI) in different *Tamyb10* genotypes in 103 germplasm

Genotype	No. of varieties	GI (%)	GI range (%)
aaa	26	26.4 ± 3.2 a	3.8–68.4
aab	12	19.8 ± 4.7 a	4.5–56
aba	24	50.8 ± 2.4 b	22.9–70.8
abb	3	27.5 ± 11.8 a	4–40.7
baa	12	28.3 ± 2.9 a	8.3–46.2
bab	5	17.2 ± 5.6 a	6.7–32.5
bba	18	51.9 ± 2.3 b	38.9–69.9
bbb	3	41.2 ± 15.7 ab	10.2–61.5

GI value is the mean ± SE of experiments in two locations over 2 years. Different lowercase letters after average GI ± SE represent significant differences (*P* < 0.01)

## Discussion

An association between grain color and depth of dormancy has been noted in a *Vp-1* mutant of maize and in transparent testa (tt) mutants of *Arabidopsis* (McCarty et al. 1991; Debeaujon et al. 2000). Normally, red-grained varieties are usually more tolerant to PHS than white-grained wheat varieties (Flintham 2000; Warner et al. 2000; Himi et al. 2002); however, it is proven that the statement is not all true in the present study. Some white-grained varieties, such as Xumai 954, Fengchan 3, Xinmai 13, Yumai 18, and Xiaoyuhua, had higher PHS tolerance with germination rates below 10 % (Table 1), although they had an allele combination of aaa. White-grained wheat varieties are homozygous for the recessive alleles of *R-1* gene, while red-grained wheat varieties are heterozygous or homozygous for the dormant alleles of *R-1* gene ((McIntosh et al. 1998), and no amplified fragments from genomic DNA and cDNA of white-grained wheats were obtained by PCR using specific primers of *Tamyb10-Da* before (Himi et al. 2011). In the present study, a part of genomic sequences of *Tamyb10-Da* (1419 bp) were amplified fortunately from bread wheat (*R-D1a* varieties) with higher GI values. Compared with the *Tamyb10-D1b* (AB191460), ORF of *Tamyb10-Da* also had very high similarity with *Tamyb10-B1a* besides sequence of AB191460. Among the 103 Chinese bread wheat varieties, only 23 (average GI value was 0.245) were the allele *Tamyb10-D1b*, and the others (average GI value was 0.398) were *Tamyb10-D1a* with only one 1419-bp fragment amplified, suggesting that mutation locus of *Tamyb10-D1a* might occur in the region between 1419 and 1629 bp, leading an early termination in the coding region; that is why the nonspecific PCR product were amplified with the primer sets Tamyb10-DF_1_/R_2_ and Tamyb10-DF_3_/R_3_ in these varieties.

Chinese spring with deletions of chromosome 3DL produces seeds with white color (Himi and Noda 2004), indicating that deletion of either the *DFR* gene or *Myb/c* or *TaVp-1D* or all of these three genes on chromosome 3D have loss or gain of functions in grain color (Xia et al. 2008); moreover, in red-grained wheat, *Tamyb10-D1* has the greatest effect on PHS resistance, followed by *Tamyb10-B1*, and *Tamyb10-A1* had the least effect (Wang et al. 2014). In this germplasm, there is no significant difference of GI between *Tamyb10-A1b* and *Tamyb10-A1a* genotypes, but for *Tamyb10-B1* and *Tamyb10-D1*, the significant difference of GI is present between *Tamyb10-B1a* and *Tamyb10-B1b* (*F* = 1.400, *P* = 0.9 × 10^−13^) and between *Tamyb10-D1b* and *Tamyb10-D1a* (*F* = 0.296, *P* = 0.00012), respectively. Moreover, varieties with allele *Tamyb10-B1a* were more resistant than that with *Tamyb10-B1b*, and the varieties with *Tamyb10-D1b* were more resistant than those with *Tamyb10-D1a*. The above data showed that *Tamyb10-B1* had the greatest effect on seed dormancy, followed by *Tamyb10-D1*, and *Tamyb10-A1* had the least effect, and these also showed that there is no correlation between the numbers of *R-b* (including *R-A1b*, *R-B1b*, and *R-D1b*) and the level of seed dormancy in a variety. It may be a reason that the red-grained wheat varieties are usually more tolerant to PHS than white-grained wheat varieties, but not all the red grained wheats had higher PHS resistance than white grained. In this study, there was another interesting result; the RIL population of Yangxiaomai/Zhongyou 9507 was also used to confirm the association of *Tamyb10-B* variations (*Tamyb10-Ba* and *Tamyb10-Bb*) and GI values, but statistical analysis showed no significant differences of GI (*F* = 6.67, *P* = 0.254) between two genotypes.

In our previous study, STS markers *Vp1A3* and *Vp1B3* were associated with seed dormancy in Chinese wheats with different GI values (Yang et al. 2007, 2014); the efficiency of marker-assisted selection for PHS-resistant varieties was improved using *Vp1A3* and *Vp1B3*. The allele combinations *TaVp-1Agm* and *TaVp-1Bb*, *TaVp-1Agm* and *TaVp-1Ba*, *TaVp-1Aim* and *TaVp-1Bb*, and *TaVp-1Aam* and *TaVp-1Bb* could confer higher PHS resistance (Yang et al. 2014). But, some varieties such as Suiningtuotuomai (average GI = 0.10) and Waitoubai (average GI = 0.07) with the alleles *TaVp-1Aam* and *TaVp-1Bc* did not carry any of the PHS-resistant allele combinations but also had the higher PHS resistance (Yang et al. 2014). Interestingly, Suiningtuotuomai and Waitoubai both carry *Tamyb10-Db* amplified with the STS marker *Tamyb10D* associated with PHS tolerance. Therefore, for PHS resistance breeding in wheat, using STS marker combinations (*Vp1A3*, *Vp1B3*, and *Tamyb10D*) will improve the efficiency of selection very much. Because *Vp1A3* and *Vp1B3* are also associated with embryo-imposed dormancy, and *Tamyb10D* is associated with coat-imposed dormancy, the mechanism of seed dormancy can be divided into embryo-imposed and coat-imposed dormancy.
